# Amifostine inhibits acrylamide-induced hepatotoxicity by inhibiting oxidative stress and apoptosis

**DOI:** 10.22038/IJBMS.2023.67815.14837

**Published:** 2023

**Authors:** Mostafa Karimi, Mahboobeh Ghasemzadeh Rahbardar, Bibi Marjan Razavi, Hossein Hosseinzadeh

**Affiliations:** 1 Department of Pharmacodynamics and Toxicology, School of Pharmacy, Mashhad University of Medical Sciences, Mashhad, Iran; 2 Pharmaceutical Research Center, Pharmaceutical Technology Institute, Mashhad University of Medical Sciences, Mashhad, Iran; 3 Targeted Drug Delivery Research Center, Pharmaceutical Technology Institute, Mashhad University of Medical Sciences, Mashhad, Iran

**Keywords:** Acrylamide, Amifostine, Apoptosis, Hepatotoxicity, Oxidative stress

## Abstract

**Objective(s):**

Acrylamide (ACR) is a toxic chemical agent that can induce hepatotoxicity through different mechanisms including oxidative stress and apoptosis. Amifostine is an important hepatoprotective and anti-oxidant compound. In this research, the hepatoprotective effect of amifostine on ACR-induced hepatotoxicity in rats has been investigated.

**Materials and Methods:**

Male Wistar rats were randomly divided into 7 groups, including: 1. Control group, 2. ACR (50 mg/kg, 11 days, IP), 3-5. ACR+ amifostine (25, 50, 100 mg/kg, 11 days, IP), 6. ACR+ N-acetyl cysteine (NAC) (200 mg/kg, 11 days, IP), and 7. Amifostine (100 mg/kg, 11 days, IP). At the end of the injection period, animals’ liver samples were collected to determine the content of glutathione (GSH), malondialdehyde (MDA), and apoptotic proteins (B-cell lymphoma 2 (Bcl2), Bcl-2-associated X protein (Bax), and cleaved caspase-3. Serum samples were also collected to measure alanine transaminase (ALT) and aspartate transaminase (AST) levels.

**Results:**

Administration of ACR increased MDA, Bax/Bcl2 ratio, cleaved caspase-3, ALT, and AST levels, and decreased GSH content compared with the control group. The administration of amifostine with ACR decreased MDA, Bax/Bcl2 ratio, cleaved caspase-3, ALT, and AST levels, and increased GSH content compared with the ACR group. Receiving NAC along with ACR reversed the alterations induced by ACR.

**Conclusion:**

This study shows that pretreatment with amifostine can reduce ACR-induced toxicity in the liver tissue of rats. Since oxidative stress is one of the most important mechanisms in ACR toxicity, amifostine probably reduces the toxicity of ACR by increasing the anti-oxidant and anti-apoptotic capacity of the hepatic cells.

## Introduction

Acrylamide (ACR) with the chemical formula of C3H5NO is a water-soluble vinyl polymer, which is used to produce poly-ACR materials. It is also used in various industrial fields, for instance, cosmetic, paper, and textile industries (1). Researchers of Stockholm University have reported that ACR is formed during cooking at high temperatures (above 120 °C) including roasting, grilling, baking, and frying carbohydrate-rich foods via interactions of reducing sugar such as glucose with amino acids like asparagine (2-4). Accordingly, individuals who consume high temperature cooked foods are estimated to take 0.5 mg/kg/day of ACR (5). Several papers have reported ACR-induced hepatotoxicity (6-8), neurotoxicity (9), nephrotoxicity (10), genotoxicity (11, 12), and carcinogenic effects (13) in rodents and humans. 

It has been observed that ACR induces oxidative stress by affecting the cellular redox chain and generating reactive oxygen species (ROS). After oxidization to glycidamide, ACR conjugates with glutathione (GSH). When ACR concentration increases, the GSH amount decreases (14, 15). In humans, oxidative stress could be induced by chronic exposure to dietary ACR. It was disclosed that after exposing isolated human monocyte to ACR, ROS formation and GSH oxidation were elevated. Furthermore, it has been illustrated that ACR can induce apoptosis due to oxidative stress (16). 

Following oral or intraperitoneal (IP) administration of ACR, the liver is the preliminary target of ACR metabolism. Thus, the liver serves as a target organ for ACR and hepatotoxicity is a prevalent reported adverse effect of ACR (17). It was stated that exposing HepG2 cells, a human liver cancer cell line, to ACR, triggers ROS generation and time and concentration-dependent GSH reduction in hepatocytes (18). In addition, some *in vitro* studies indicated that ACR increases the amount of lipid peroxidation and declines the GSH level in the liver (19, 20). Some other research projects have revealed that ACR exposure to rodents and humans increases the amount of caspase-3 (the final mediator of the apoptosis signaling pathway) and the Bcl-2-associated X protein (Bax)/B-cell lymphoma 2 (Bcl2) ratio (8, 21, 22). Furthermore, it has also been observed that ACR administration increases the aspartate aminotransferase (AST) and alanine aminotransferase (ALT) amount (23, 24).

It is reported that the effective treatment or prevention of the mentioned ACR-induced adverse effects depends on anti-oxidant, anti-apoptotic, and hepatoprotective potential agents (11, 25, 26).

Amifostine which is a thiophosphate derivative of cysteamine could be prescribed before cytotoxic chemotherapy to protect normal tissues, without weakening antitumor response (27, 28). Amifostine converts to its active metabolite, WR-1065, in the plasma membrane via dephosphorylation by alkaline phosphatase. When WR-1065 enters the cell, its protective properties reveal by producing mixed disulfides to defend normal cells against cytotoxic chemotherapy compounds, scavenging free radicals, donating hydrogen, and liberating endogenous nonprotein sulfhydryls, principally GSH, from their bonds with proteins (28, 29).

Studies have shown that the metabolite WR-1065 can provide cellular protection by sweeping radiation-induced oxygen free radicals and inhibiting nucleophiles that may cause cross-linking and deoxyribonucleic acid (DNA) damage (30-32). Research has suggested that the active metabolite of amifostine may increase the expression of the P53 gene, leading to cell cycle accumulation in the gap 1- synthesis (G1-S) phase, which may provide a greater opportunity for adequate DNA repair. Extensive *in vitro* and *in vivo* preclinical studies have shown that amifostine selectively protects healthy tissue, whereas, in a wide range of human and mouse carcinomas, sarcomas, and leukemias it has no protective effects (33-35).

According to previous studies on the protective effects of amifostine, in this study, the possible protective effects of amifostine (25, 50, 100 mg/kg, IP) on ACR (50 mg/kg, IP) toxicity on male Wistar rats were investigated during 11 days by assessing lipid peroxidation (malondialdehyde (MDA), GSH) in liver tissue, serum biochemical factors (ALT, AST), and apoptotic factors (Bax, Bcl2, and cleaved caspase-3) in liver tissue. 

## Materials and Methods

***Animals***

In this study, male Wistar rats weighing 200 to 210 g bred in the animal room of Mashhad School of Pharmacy were used. These animals were kept in the temperature range of 22–25 °C in the light condition of 12 hr of darkness and 12 hr of light. There was no restriction on access to water and food. The experiments were approved by the Animal Care and Use Committee of Mashhad University of Medical Sciences, Iran (ethical number: IR.MUMS.SP.1396.129) and carried out consistent with internationally accepted principles for animal use and care (36).

***Materials***

Xylazine and ketamine were acquired from Alfasan Pharmaceutical Co, Woerden, the Netherlands. N-acetylcysteine (NAC) (positive reference drug) was obtained from Exir company, Iran. Amifostine was purchased from Well Stone, China. ACR was bought from Sigma, Germany.

***Study protocol***

Thirty five male Wistar rats were randomly divided into 7 groups (n=5):

1- Rats receiving vehicle (normal saline, IP);

2- Rats receiving ACR (50 mg/kg, IP) (37);

3- Rats receiving ACR (50 mg/kg, IP) + amifostine (25 mg/kg, IP);

4- Rats receiving ACR (50 mg/kg, IP) + amifostine (50 mg/kg, IP);

5- Rats receiving ACR (50 mg/kg, IP) + amifostine (100 mg/kg, IP) (38);

6- Rats receiving amifostine (100 mg/kg, IP);

7- Rats receiving ACR (50 mg/kg, IP) + NAC ​​(200 mg/kg, IP) (39).

According to the classification, pretreatment of some groups was performed by amifostine and NAC for three days, and from the fourth day each rat, based on its weight, received ACR and hepatoprotective substance daily related to its group until the eleventh day (17, 40). Other injections were given for eleven days. At the end of the administration period, the animals were sacrificed in deep anesthesia using ketamine (60 mg/kg) and xylazine (6 mg/kg) intraperitoneally, and their liver tissue and blood samples were isolated for further tests.

***Measuring MDA amount in the liver tissue***

The MDA measurement method is based on the color spectrophotometric extent of the reaction of thiobarbituric acid (TBA) with MDA in an acidic environment and the formation of pink color. An increase in pink color is a sign of a rise in lipid peroxidation. This pink complex has a maximum absorption at 532 nm (41).

The isolated tissues were taken out from the freezer at -80 °C and kept in liquid nitrogen until the beginning of the experiment. A homogeneous tissue sample of 10% was prepared in 1.15% cold KCl by a homogenizer and stored on ice. Then 3 ml of phosphoric acid and 1 ml of TBA were added to 500 µl of homogenized tissue and then the resulting mixture was placed in boiling water for 45 min. Four ml of n-butanol was then added to the cooled mixture and vortexed for one minute. Then it was centrifuged with 3000 g for 15 min and then the organic phase was isolated, and the absorption rate of this phase was measured in a spectrophotometer with a wavelength of 532 nm for different samples. To calculate the MDA concentration, the standard curve was plotted in the range of 0-100 nmol/ml MDA concentrations and finally, the concentrations were reported in terms of nmol/g tissue.

***Measuring GSH amount in the liver tissue***

This method is based on the reaction of free sulfhydryl groups with 5,5′-dithiobis-(2-nitrobenzoic acid) (DTNB) reagent in an alkaline medium. The resulting color complex has a maximum absorption of 412 nm. GSH is used to draw the standard curve (42).

The isolated tissues were taken out from the freezer at -80 °C and kept in liquid nitrogen until the initiation of the experiment. 10% homogeneous tissues were prepared in phosphate buffer (pH 7.4). The homogenized tissue was mixed (1:1) with (10%) trichloroacetate (TCA) and then centrifuged at 3000 g for 10 min. Then 500 µl of the supernatant was mixed with 2.5 ml of phosphate buffer (pH 8) and 500 µl of DTNB reagent (0.04% in 10% sodium citrate) and then the absorbance was measured at 412 nm in spectrophotometry. To calculate the GSH concentration, a standard curve was drawn in the range of 0-300 nmol/ml of GSH concentrations and finally, the concentrations were reported in terms of nmol/g tissue.

***Western blotting***

After unfreezing, the samples of liver were put in a lysis buffer containing 50 mM Tris–HCl (pH: 7.4), 2 mM ethylenediaminetetraacetic acid (EDTA), 2 mM egtazic acid (EGTA), 10 mM NaF, 1 mM sodium orthovanadate (Na_3_VO_4_2H_2_O), 10 mM βglycerophosphate, 0.2% W/V sodium deoxycholate, 1 mM phenylmethylsulfonyl fluoride, and complete protease inhibitor cocktail (Roche, Mannheim, Germany). The homogenates were then sonicated on ice with three 10 sec bursts at high intensity with a 10 sec cooling period between each and centrifuged (10,000g) for 10 min at 4 °C. To evaluate protein concentration and regulate the contents of samples, the Bradford assay kit (Bio-Rad; Bradford, 1976) was applied. Each adjusted sample was mixed 1:1 v:v with 2× sodium dodecyl sulfate (SDS) blue buffer, boiled, aliquoted, and kept in a −80 °C freezer. Samples were loaded (50 μg of protein/lane), electrophoresed on a 12% SDS-polyACR gel electrophoresis (SDS = PAGE), and blotted to a polyvinylidene fluoride membrane (PVDF; Bio-Rad). Then, the PVDF papers were blocked in skim milk for 2 hr. The blots were incubated for 2 hr on a rocker with rabbit polyclonal anti-Bax (Cell Signaling #2772, 1:1,000), rabbit polyclonal anti-Bcl2 (Cell Signaling #2870, 1:1,000), anti-cleaved caspase-3 (Cell Signaling #9664, 1:1,000), and mouse polyclonal anti-β-actin antibodies (Cell Signaling #4967, 1:1,000). After washing three times with Tris-Buffered Saline and Tween 20 (TBST), membranes were incubated with rabbit or mouse horseradish peroxidase-conjugate anti-IgG (Cell Signaling #7071, 1:2,000; Cell Signaling #7072, 1:2,000, respectively) for 2 hr. Enhanced chemiluminescence (ECL) was used to visualize the peroxidase-coated bands. The integrated optical densities of bands were measured using Alliance 4.7 Gel doc (UK). Densitometric analysis for protein bands was performed using UV Tec Software (UK). The protein levels were normalized relative to the corresponding bands of β-actin as a control protein.

***Statistics***

Statistical calculations were performed using the Prism 6 software program. The results are shown as Mean±SEM. For comparison between different groups in the study, ANOVA and Tukey-Kramer post-test were used and a *P*-value less than 0.05 was considered a significant difference. 

## Results

***Effect of amifostine and ACR on MDA level in liver tissue***

**Figure 1:**
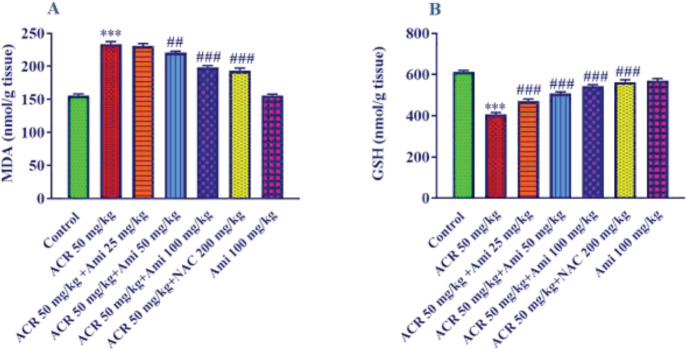
Effect of amifostine and ACR on A: MDA and B: GSH levels in liver tissue. Data is displayed as SEM±Mean (n=5). One-way ANOVA test and Tukey-Kramer post-test were used to investigate the statistical difference. ## *P*<0.01 and ### *P*<0.001 compared with ACR group and *** *P*<0.001 compared with the control group

The results show that the amount of MDA in the ACR-receiving group increased significantly compared with the control group (*P*<0.001). MDA levels in the liver of pre-treated rats at doses of 50 and 100 mg/kg of amifostine were significantly reduced compared with the ACR alone group (*P*<0.01 and *P*<0.001, respectively). Also, the use of NAC at a dose of 200 mg/kg with ACR significantly reduced the amount of MDA in liver tissue (*P*<0.001). MDA levels in rats receiving 100 mg/kg amifostine alone did not show a significant change compared with the control group (Figure 1A).

***Effect of amifostine and ACR on GSH level in liver tissue***

Reduced GSH levels indicate anti-oxidant capacity. The results showed that ACR (50 mg/kg) significantly decreased the amount of GSH compared with the control group (*P*<0.001) (Figure 1B). Pretreatment of rats with amifostine (25, 50, and 100 mg/kg) and NAC (200 mg/kg) significantly increased the level of GSH compared with the ACR alone group (*P*<0.001).

***Effect of amifostine and ACR on AST and ALT levels in liver tissue***

**Figure 2:**
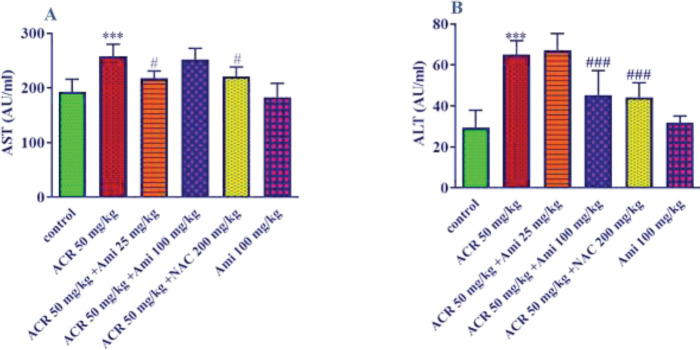
Effect of amifostine and ACR on A: AST and B: ALT levels in liver tissue. Data is displayed as SEM±Mean (n=5). One-way ANOVA test and Tukey-Kramer post-test were used to investigate the statistical difference. # *P*<0.05 and ### *P*<0.001 compared with the ACR group, and *** *P*<0.001 compared with the control group

ACR: Acrylamide; Ami: Amifostine; NAC: N-acetyl cysteine

The results showed that ACR (50 mg/kg) caused a significant increase in ALT and AST levels in the liver of animals compared with the control group (*P*<0.001). On the other hand, amifostine (25 mg/kg) was able to prevent the increase in AST levels caused by ACR (*P*<0.01) (Figure 2A). In addition, amifostine (100 mg/kg) could significantly prevent the ALT elevation induced by ACR (*P*<0.001). It was also observed that NAC (200 mg/kg) significantly reduced the amounts of ALT (*P*<0.001) and AST (*P*<0.05) compared with the ACR alone group (Figure 2A, B). 

***Effect of amifostine and ACR on Bax/Bcl2 ratio in liver tissue***

According to the results of MDA and GSH, amifostine (100 mg/kg) was selected to evaluate the amount of proteins involved in apoptosis.

**Figure 3:**
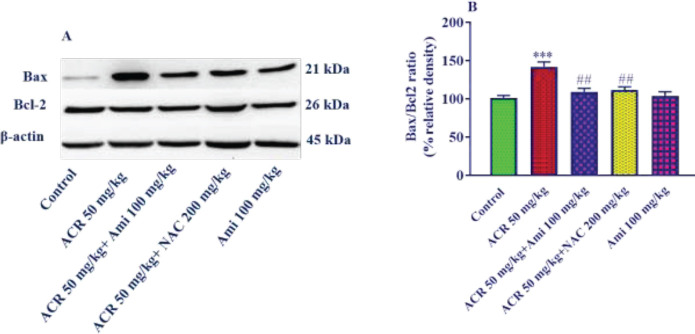
Effect of amifostine and ACR on Bax/Bcl2 ratio in liver tissue. A: Represent immunoblot bands of the western blotting analysis; B: Represent quantitative presentation of the immunoblots. Data is displayed as SEM±Mean (n=5). One-way ANOVA test and Tukey-Kramer post-test were used to investigate the statistical difference. ## *P*<0.01 compared with the ACR group and *** *P*<0.001 compared with the control group

ACR: Acrylamide; Ami: Amifostine; NAC: N-acetyl cysteine

The results of the Bax/Bcl2 ratio analysis showed that this ratio increased significantly in the ACR-receiving group compared with the control group (*P*<0.001). It was also observed that pretreatment with amifostine (100 mg/kg) and NAC (200 mg/kg) significantly reduced the ratio of Bax/Bcl2 (*P*<0.01) compared with the ACR alone group (Figure 3).

***Effect of amifostine and ACR on the expression of cleaved caspase-3 protein in liver tissue***

**Figure 4:**
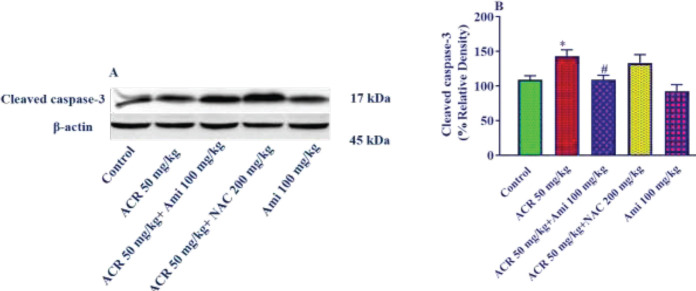
Effect of amifostine and ACR on cleaved caspase-3 protein expression in liver tissue. A: Represent immunoblot bands of the western blotting analysis; B: Represent quantitative presentation of the immunoblots. Data is displayed as SEM±Mean (n=5). One-way ANOVA test and Tukey-Kramer post-test were used to investigate the statistical difference. # *P*<0.05 compared with the ACR group and * *P*<0.05 compared with the control group

ACR: Acrylamide; Ami: Amifostine; NAC: N-acetyl cysteine

The data revealed that the cleaved caspase-3 protein level significantly increased in ACR (50 mg/kg) group compared with the control group (*P*<0.05) (Figure 4). It was also seen that the expression of this protein significantly decreased in the amifostine (100 mg/kg) plus ACR group compared with the ACR alone group (*P*<0.05). Moreover, the administration of NAC (200 mg/kg) reduced cleaved caspase-3 protein level but it was not significant.

## Discussion

In the present work, the effect of amifostine (25, 50, and 100 mg/kg, IP) on ACR (50 mg/kg, IP)-induced hepatotoxicity in rats was assessed during 11 days. It was observed that ACR (50 mg/kg) increased MDA, Bax/Bcl2 ratio, and cleaved caspase-3 in the liver tissue, decreased GSH amount in the liver tissue, and boosted ALT and AST amount in serum. These alterations were almost amended by the administration of different doses of amifostine and NAC (200 mg/kg, IP), as a reference drug. 

The most sensitive and widely used diagnostic enzymes in the liver are aminotransferases. These enzymes include aspartate aminotransferase (SGOT or AST) and alanine aminotransferase (SGPT or ALT). These enzymes are normally located inside the liver cells. When the liver is damaged, the liver cells release enzymes into the bloodstream (43, 44).

The obtained data displayed that ACR (50 mg/kg) caused a significant increase in ALT and AST levels in the liver of animals compared with the control group. In other words, ACR caused liver damage in animals, which is characterized by an increase in liver enzymes. Although different doses of amifostine decreased AST levels, this decrease was significant only at the dose of 25 mg/kg, and amifostine (100 mg/kg) was able to decrease the ALT level. Considering that ACR caused a 34% and 124% increase in AST and ALT levels, respectively, it may explain why the low dose of amifostine did not ameliorate the ACR-induced elevation in ALT levels.

In former research, the hepatotoxicity of ACR has been investigated by applying different concentrations of ACR on liver cells, and the levels of ALT and AST leakage were measured. The results show that ALT and AST leakage increased significantly after 30 min of exposure to ACR compared with the control group (45). The results of another study showed that the administration of ACR (40 mg/kg, 10 days) to male rats increased serum levels of ALT and AST (26).

Furthermore, it was observed that prescribing amifostine (50 mg/kg) could decrease ALT and AST levels in methotrexate-induced hepatotoxicity in rats (46). The hepatoprotective property of amifostine nanoemulsion (SiNPs@AMF) was also illustrated in cisplatin hepatotoxic rats (47).

The results of lipid peroxidation in liver tissue showed that receiving ACR (50 mg/kg) significantly increased the MDA level and decreased the GSH amount compared with the control group. The results also showed that amifostine ameliorated these induced alterations. 

Consistent with our outcomes, it was observed that the administration of ACR (25 mg/kg/day, gavage) to rats increased MDA and reduced GSH amounts in the liver (25). Also, in a study by Stankiewicz and colleagues, the effects of amifostine on cyclophosphamide-induced oxidative stress in rat liver tissue were investigated. This study showed that intraperitoneal administration of cyclophosphamide decreased the activities of liver anti-oxidant enzymes such as superoxide dismutase, glutathione peroxidase, and glutathione reductase. In this study, the co-administration of amifostine and cyclophosphamide significantly increased the activity of these enzymes, including glutathione reductase (48). Another research on adriamycin-induced cardiotoxicity in rats claimed that intraperitoneal administration of amifostine (200 mg/kg) 30 min before administration of adriamycin decreases lipid peroxidation by decreasing MDA and increasing GSH levels in cardiac tissue (49).

Studies have shown that Bax and Bcl2 are two primary members of the Bcl2 family that affect mitochondrial membrane permeability. Bax is a pro-apoptotic protein that accelerates cell apoptosis, but Bcl2 has anti-apoptotic properties (50, 51). The balance between pro-apoptotic and anti-apoptotic proteins of the Bcl2 family is important in the progression of apoptosis, so the Bax/Bcl2 ratio is considered an important factor in predicting apoptosis (52).

The results of this study showed that intraperitoneal administration of ACR (50 mg/kg) for 11 days caused a significant increase in Bax protein expression and a significant increase in the Bax/Bcl2 ratio in the liver tissue of the rats. The results also disclosed that pretreatment with amifostine (100 mg/kg) significantly reduced Bax expression and Bax/Bcl2 ratio. The results of the evaluation of cleaved caspase-3 showed that the level of expression of this protein in the ACR group significantly increased compared with the control group. On the other hand, pretreatment of animals with amifostine (100 mg/kg) significantly reduced the expression of this protein compared with the ACR group.

In a study, the mechanism of cytotoxic effects of ACR in isolated rat liver was investigated and animals were exposed to 1 mM ACR for 2 hr by hepatic perfusion. The outcomes illustrated that ACR caused the formation of ROS and lipid peroxidation. In addition, the results showed that ACR increased the activity of caspase-3 as the last mediator in signaling apoptosis (21). In an *in vivo* study, Lakshmi *et al*. examined the effects of fish oil on oxidative stress and induction of ACR-induced apoptosis in the rat cerebral cortex. In this study, rats received ACR (30 mg/kg) intraperitoneally for 30 days. The results of this study showed that the administration of ACR significantly reduced Bcl2 expression and increased Bax compared with the control group (53). On the other hand, the results of another research showed that amifostine (70 mg/kg, IP) can suppress p53 and Bax and reduce caspase-3 activation. In this study, female mice were exposed to gamma radiation (6.42 Gy). It was observed that in the groups exposed to radioactivity, caspase-3, and PARP had converted to their active forms, and in the pretreatment groups with amifostine, the increase in p53 and Bax was suppressed and the decrease in the level of the active form of caspase-3 showed that amifostine was able to stop cell death due to its anti-apoptotic effects (53).

In the current work, NAC was selected as a positive control group (reference drug) because there are structural and functional similarities between this substance and amifostine and both of them are cytoprotective anti-oxidants (46). It was observed that NAC (200 mg/kg, IP) decreased MDA, AST, ALT, and Bax/Bcl2 ratio, and increased GSH. In line with our data, another study concluded that NAC (20 mg/kg, oral, for 6 weeks) treatment could attenuate oxidative stress and improve liver histology in rats with non-alcoholic steatohepatitis (54). In another study, it was illustrated that NAC protects against liver injury induced by carbon tetrachloride by decreasing ALT, AST, and MDA levels (55). It was reported that NAC (10 mM, 12 months, p.o.) administration to mice attenuates hepatocyte damage and apoptosis by decreasing the amount of caspase-3 (56). Another study has also shown that receiving NAC (80 mg/kg, p.o.) for 7 weeks reduced caspase-3 levels and protects against nonalcoholic fatty liver disease in young rats (57). Therefore, it might be proposed that NAC needs a longer time to affect the amount of caspase-3 and that is why NAC did not reduce the level of cleaved-caspase-3 in our experiment. 

The findings of the current work demonstrated that amifostine (100 mg/kg, IP) and NAC (200 mg/kg, IP) were equally effective in reducing oxidative stress, apoptosis, and increasing the function of the liver tissue.

## Conclusion

In brief, the results reveal that lipid peroxidation and apoptosis signaling pathways are implied in ACR-induced hepatotoxicity in rats. ACR induces oxidative stress and apoptosis, indicated by a rise in MDA amount, a reduction in GSH level, an enhancement in Bax/Bcl2 ratio, and cleaved caspase-3 protein level. Consequently, amifostine has hepatoprotective properties against ACR-induced hepatotoxicity in rats by inhibiting oxidative stress and apoptosis.

## Authors’ Contributions

HH and BMR conceived the study, conducted the work, and supervized; MK did the experiments and the statistical analysis; MGR wrote the manuscript.

## Ethical Approval

All animal experimental protocols were approved by the Animal Ethics Committee of Mashhad University of Medical Sciences, Iran.

## Conflicts of interest

The authors declare that they have no conflicts of interest.
